# Evaluation of RANKL/OPG Serum Concentration Ratio as a New Biomarker for Coronary Artery Calcification: A Pilot Study

**DOI:** 10.1155/2012/306263

**Published:** 2012-03-28

**Authors:** Amir Hooshang Mohammadpour, Jamal Shamsara, Saeed Nazemi, Samira Ghadirzadeh, Shabnam Shahsavand, Mohammad Ramezani

**Affiliations:** ^1^Department of Pharmacodynamy and Toxicology, School of Pharmacy, Mashhad University of Medical Sciences, Mashhad 9188865531, Iran; ^2^Pharmaceutical Research Center, Mashhad University of Medical Sciences, P.O. Box 91775-1365, Mashhad 9188865531, Iran; ^3^Department of Biotechnology, School of Pharmacy, Mashhad University of Medical Sciences, Mashhad 9188865531, Iran; ^4^Department of Cardiovascular Diseases, Razavi Hospital, 9148857114, Iran; ^5^Nanotechnology Research Center, Mashhad University of Medical Sciences, Mashhad 9188865531, Iran

## Abstract

*Objective*. There is a strong need for biomarkers to identify patients at risk for future cardiovascular events related with progressive atherosclerotic disease. Osteoprotegerin (OPG) protects the skeleton from excessive bone resorption by binding to receptor activator of nuclear factor-*κ*B ligand (RANKL) and preventing it from binding to its receptor, receptor activator of nuclear factor-*κ*B. However, conflicting results have been obtained about association of serum level of OPG or RANKL with coronary artery disease (CAD). Based on their role in inflammation and matrix degradation and the fact that atherosclerotic plaque formation is an inflammatory process, we hypothesized that RANKL : OPG ratio could be a better biomarker for CAD. *Methods*. In this cross-sectional study, the correlation between RANKL : OPG ratio serum concentration and coronary artery calcification (CAC) in 50 patients with ischemic coronary disease has been investigated. We used ELISA method for measuring RANKL and OPG serum concentrations. *Results*. There was a significant correlation between RANKL : OPG serum concentration ratio and CAC in our study population (*P* = 0.01). *Conclusion*. Our results suggested that RANKL : OPG ratio concentration has a potential of being used as a marker for coronary artery disease.

## 1. Introduction

There is a considerable demand for diagnosis and treatment of the pathologic conditions that underlie sudden cardiac events such as acute coronary syndromes and sudden cardiac death. Some people experience a cardiovascular event, while, by Framingham scores, they were not considered to be at high-risk group [1, 2]. Atherosclerotic calcification is a failure that can make the break in the vessels and cause the plaque rupture [3, 4]. Plaque rupture is the most common type of plaque complications. Several retrospective autopsy series and a few cross-sectional clinical studies have suggested that thrombotic coronary death and acute coronary syndromes are caused by the plaque features [5, 6]. This phenomenon is a regular and organized process that is so similar to osteoproduction [7]. Some of the smooth muscle cells along the migration from layer to vascular media layer go osteogenic [8]. These cells continuously express osteoproteins such as RANKL and OPG [4] and start to store the collagenic extracellular matrix in mineralized collections that finally cause vascular calcification [9]. RANKL/RANK/OPG system has an important role in several aspects of the processes leading to calcification [3]. RANKL binds to its membrane receptor RANK and produces several intracellular signals that regulate the fusion, development, function, and survival of the osteoclasts [2, 3]. It also stimulates the gradual development of osteogenic calcification in the vascular smooth muscle cells [10]. OPG as a soluble scavenger presents the RANKL/RANK binding so it inhibits the RANKL function [3]. It has inhibitory effects on osteoclastogenes and osteogenic resorption [2]. Some reports indicated that, in cardiovascular system, serum concentration of OPG increases in clinical cases susceptible to atherosclerosis and unstable vascular calcification [3] OPG secretion probably is an imperfect compensatory mechanism in response to increase of RANKL secretion that could prevent calcification and atherosclerosis [11]. So increased RANKL concentration and decreased OPG level both can lead to vascular calcification. According to this, we evaluated the RANKL/OPG ratio as a diagnostic biomarker to determine the extent of vascular calcification and subsequent coronary disorders such as coronary artery calcification (CAC).

## 2. Patients and Methods

### 2.1. Patients

Fifty patients with ischemic coronary disease (37 men and 13 women, age 18–60) were enrolled in this study between November 2008 and September 2009. Patients were from Razavi Hospital in Mashhad. Patients with calcium and phosphorous metabolic failure, parathyroid disease, renal dysfunction, history of osteodisorder, zero calcium score, and vit D consumption were excluded from the study. A questionnaire containing demographic data, laboratory data, drug history, medical history, familial history of CV risk factors was completed for all patients. All patients signed the consent form prior to entry in the study.

### 2.2. Blood Sampling and Biochemical Assay

Whole blood was collected from patients and centrifuged at 2500 rpm for 10 min. The plasma fraction was isolated and stored at −70°C until required for analysis. Routine biochemical measurements such as plasma glucose, total cholesterol (TC), triglycerides, low density lipoprotein cholesterol (LDL), high-density lipoprotein cholesterol (HDL), serum calcium, phosphorus were carried out by routine laboratory methods. 

### 2.3. Determination of OPG and RANKL Serum Concentration

Serum level of soluble OPG and RANKL was measured with an enzyme-linked immunosorbant assay (ELISA) kit (Apotech, Switzerland); each assay was calibrated using RANKL and OPG standard curve following the manufacturer's instructions.

### 2.4. Statistical Analysis

Statistical analysis was carried out by SPSS 11.5. Correlation between serum concentration of RANKL, OPG, and RANKL/OPG ratio with CAC (coronary artery calcification) was analyzed using Pearson correlation. To compare serum concentration of RANKL, OPG, and RANKL/OPG ratio between different groups, one-way ANOVA test was used. Results were considered significant at *P* < 0.05. All measured values are presented as the mean ± SD. 

## 3. Results 

### 3.1. Characteristics of the Study Population

Demographic data, biochemical data (laboratory tests) including OPG and RANKL levels, and traditional cardiovascular risk factors are summarized in Table 1.

### 3.2. Correlation between RANKL : OPG Ratio and Total Coronary Artery Calcification

**Figure 1:**
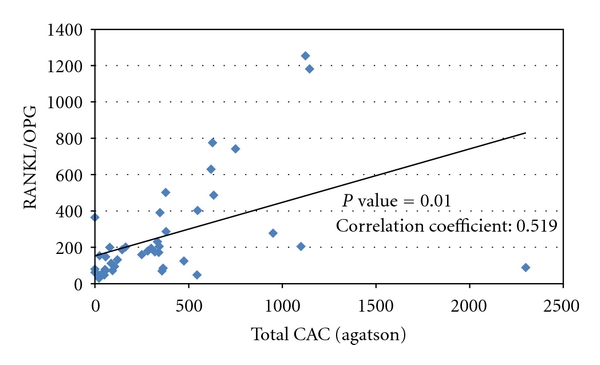
Correlation between RANKL : OPG ratio and coronary artery calcification.

There was a significant positive correlation between RANKL : OPG ratio and total coronary artery calcification (Table 2 and Figure 1) and negative correlation between OPG and total coronary artery calcification (Table 2). There was no significant correlation between RANKL serum concentration coronary artery calcification (Table 2). 

## 4. Discussion 

RANKL : OPG ratio showed stronger correlation with CAC than either RANKL or OPG concentration. 

Presence and extent of vascular calcification are mainly related to incidence of cardiovascular disorders [11]. RANKL is expressed and secreted in the semisteoblastic cells in atherosclerotic lesions and causes the gradual development of vascular osteogenic calcification [12]. OPG concentration also increases in unstable vascular calcifications and other vascular disorders. Although OPG can prevent the effects of RANKL by anatomization, maybe this increment in OPG serum level, as a protective, is not enough to neutralize the RANKL effects [13, 14]. There are several reports that indicated the relationship between OPG and RANKL effects and vascular calcification [15]. Some studies have shown that OPG can protect large blood vessels from arterial calcification based on the observation of renal and aortic calcifications occurring in OPG knockout mice [16]. Intravascular administration of OPG could prevent the induced calcification by high dose of vit D in rats [17]. It is considered that the serum concentration of RANKL was in the highest level in acute vascular syndromes such as acute myocardial infarction and ischemic cerebral vascular attacks [18]. All of previous studies just evaluated the RANKL or OPG, almost OPG, effects on development of CAC. As these factors, RANKL, RANK, and OPG, are members of one system and strongly associated, the previous results are conflicting. Some studies reported the increased serum level of OPG in CAC, while there are other evidence of inhibitory effects of OPG on vascular calcification [19]. Therefor, it is considered that evaluation of any of these factors alone cannot be useful as a diagnostic biomarker. Our study for the first time determined the changes in RANKL and OPG levels concomitantly and reported the results as RANKL/OPG ratio. As we reported, there is no significant relation between RANKL serum levels and CAC (*P* = 0.2) but there is significant negative relation between OPG serum (*P* = 0.03, CC = −0.468) and CAC and significant positive relation between RANKL/OPG ratio and CAC. It is important to mention that, in this study, measurement of OPG and RANKL level was performed in stable coronary disorders such as stable angina. In these patients, the less serum OPG levels are, the more calcification intensity occurred; this indicates the protective effects of OPG in vascular calcification. Other reports about these two factors belonged to researches on coronary syndromes such as acute myocardial infarction; therefore, it seems that the results of these two types of studies are not comparable. According to these facts, it is concluded that, in cardiovascular events, OPG serum level as a preventive compensatory mechanism markedly increased but this is not enough to neutralize the RANKL level increment. However, as a main limitation of the cross-sectional design, the RANKl/OPG ratio assessed together with the CAC is not necessarily this occurring at the same time. In conclusion, our study showed that determination of the RANKl/OPG ratio in compare with each of these two factors alone is a better diagnostic indicator for intensity of vascular calcification that leads to coronary disorders such as CAC. 

## Figures and Tables

**Table 1:** Demographic data, laboratory tests, and traditional cardiovascular risk factors of patients.

Age (year)	56.52 ± 11.05
Female/male ratio	0.35
Mean BMI	28.21 ± 4.69
*Laboratory test*	
Calcium (ng/dL)	9.02 ± 2.14
Phosphorous (ng/dL)	3.78 ± 1.35
HDL cholesterol (mg/dL)	43.95 ± 14.1
LDL cholesterol (mg/dL)	97.87 ± 35.4
Total cholesterol (mg/dL)	171.72 ± 40.63
FBS (mg/dL)	103.28 ± 30
Mean concentration of RANKL (pg/mL) (CV)	687.14 ± 114.23 (0.16)
Mean concentration of OPG (pg/mL) (CV)	6.03 ± 0.786 (0.13)
RANKL/OPG ratio (CV)	267.86 ± 31.23 (0.12)
Total calcification of coronary vessels (agatson)	356.27 ± 44.13
Calcification in coronary LAD (agatson)	234.17 ± 32.96
Calcification in coronary RCA (agatson)	63.885 ± 10.92
Calcification in coronary CX (agatson)	49.09 ± 10.59
Calcification in coronary LM (agatson)	8.49 ± 2.55
*Traditional cardiovascular risk factors*	
Hypertension (%)	54.84
Dyslipidemia (%)	73
Positive family history (%)	46
Diabetes (%)	17
Current smoking (%)	13

**Table 2:** Correlation between RANKL : OPG ratio, serum concentration of OPG, and RANKL with LAD, RCA, LMCA, and Cx coronary artery calcification.

	Serum RANKL concentration		Serum OPG concentration		RANKL/OPG	
	*P* value^1^	CC^2^	*P* value	CC	*P* value	CC
Total CAC^3^	0.2	−0.214	**0.03**	**−0.468**	**0.01**	**0.519**
CAC of LAD	0.39	−1.44	**0.013**	**−0.4**	**0.001**	**0.545**
CAC of RCA	0.17	−0.227	**0.033**	**−0.35**	0.16	0.234
CAC of LMCA	0.71	−0.62	0.42	−0.135	0.73	0.058
CAC of CX	0.21	−0.208	0.07	−0.298	0.21	0.209

^1^Pearson correlation test, ^2^Correlation coefficient, ^3^Coronary artery calcification.
